# The Isolation of Lead-Tolerant PGPR from Red Clover Soil and Its Role in Promoting the Growth of Alfalfa

**DOI:** 10.3390/microorganisms13010210

**Published:** 2025-01-19

**Authors:** Wanting Nie, Yuchen Wu, Jingwen Jiang, Zicheng Wang, Meiqi Mu, Siwen Zhao, Minghao Yang, Xi Long, Xiujie Yin, Xiaohua Teng

**Affiliations:** College of Animal Science and Technology, Northeast Agricultural University, Harbin 150030, China; m15046823374@163.com (W.N.); wuyuchen202211@163.com (Y.W.); jjw990824@163.com (J.J.); fzqwzc@163.com (Z.W.); mmq0616@163.com (M.M.); zhaosiwen2023@163.com (S.Z.); 13763669989@163.com (M.Y.); long18983055216@163.com (X.L.)

**Keywords:** abiotic stress, trace metal pollution, alfalfa, bioremediation

## Abstract

Alfalfa (*Medicago sativa* L.) is an outstanding species used for the remediation of heavy metal-contaminated soil, and our previous research has shown that PGPR can promote plant growth under high-concentration lead stress. This discovery has forced scientists to search for PGPR strains compatible with alfalfa to develop an innovative bioremediation strategy for the remediation of lead-contaminated soil. This study used lead-tolerant rhizosphere soil of red clover as experimental material; cultured, isolated, and screened 52 excellent lead-tolerant bacteria that promote rhizosphere growth; and then inoculated them into alfalfa. Marked differences existed in the secretion of auxin, protease, and ACC deaminase among these strains. The results indicated that *Pseudomonas* spp. (strain Y2), *Pseudomonas* spp. (strain Y22), and *Bacillus* spp. (strain Y23) exhibited a strong growth-promoting ability in alfalfa, and there was no antagonistic reaction among the three strains, enabling their coexistence. The pot experiment manifested that strains Y2, Y22, Y23, and YH (a mixture of Y2, Y22, and Y23) could increase the plant height, root length, fresh and dry weight above ground, and fresh and dry weight below ground of alfalfa. They could all significantly raise the chlorophyll content and antioxidant enzyme activity in alfalfa (*p* < 0.05) and the content of malondialdehyde (MDA) in alfalfa. Furthermore, the concurrent inoculation of three distinct types of plant growth-promoting rhizobacteria (PGPR) significantly diminished lead (Pb) concentrations in rhizosphere soil, enhanced the levels of available potassium (AK) and available phosphorus (AP), and augmented the capacity of plants to absorb Pb. The results imply that PGPR can be employed to facilitate plant growth and microbial-assisted remediation of lead and other heavy metal-contaminated soil and establish a basis for further research on the growth-promoting mechanism of PGPR in plants.

## 1. Introduction

In the wake of the rapid advancement of industry and the extensive application of pesticides and fertilizers, lead pollution has raised the risks associated with agricultural production activities. When lead enters the soil, the majority of it gets adsorbed and retained; yet, under specific circumstances, it can be taken up by plants and thereby enter the plant structure [1,2,3]. Once lead enters the plant via the root system, the plant will translocate it to various tissues and organs and accumulate it continuously in the diverse tissues and organs of the plant. Subsequently, this inhibits the growth and development of roots, stems, leaves, and other components of the plant, generates toxic effects for the plant, and, in severe cases, leads to the plant’s death. The higher the level of lead stress is, the more severe the phenotype damage becomes [4].

In the rhizosphere indigenous bacterial community of plants, there exists a beneficial type of bacteria that can provide nutrients for plants, promote their growth and development, and reduce the occurrence of plant diseases and pests. This type of microorganism is known as plant growth-promoting rhizobacteria (PGPR) [5]. Currently, there are numerous common types of plant-growth-promoting Rhizobacteria, mainly including *Pseudomonas* spp., *Bacillus* spp., *Enterobacter* spp., *Burkholderia* spp., and *Agrobacterium* spp., etc. [6,7]. To promote the development of plant roots and reduce the toxicity of trace metals, adding PGPR can reduce the damage to plants under biotic and abiotic stress, accelerate the efficiency of plant restoration through synergy with plants, and improve soil quality [8]. The rhizosphere bacteria successfully established in the soil ecosystem have high environmental adaptability and metabolic diversity and promote the growth of the host through symbiosis with plants. Due to the interaction between the strain and the host, PGPR promotes plant growth through various mechanisms, such as increasing phosphate solubility or phosphate biological availability, potassium dissolution, nitrogen fixation, iron carrier production, 1-aminocyclopropane-1-carboxylic acid deaminase (ACC) production, phytohormone production, the induction of systemic tolerance, and plant-growth-promoting rhizobacteria PGPR symbiosis [9]. These rhizosphere growth-promoting bacteria can not only improve resource utilization but also convert some unavailable resources of plants into available resources through their various secretions, thereby alleviating the biotic and abiotic stress on plants [10]. Mirzaei et al. investigated the drought tolerance of lemongrass (*Cymbopogon citratus* (DC.) Stapf) and discovered that PGPR inoculation could enhance the plant height; biomass; activities of SOD, POD, CAT, and other antioxidant enzymes of lemongrass under drought stress; and improve its drought tolerance [11]. Perruzza L et al. identified that *Shigella flexneri* with the apy gene encoding adenosine triphosphate phosphohydrolase could transform phosphorus in the soil that could not be directly absorbed and utilized by plants into phosphorus that could be absorbed by plants [12]. It is notable that PGPR can also supply the iron necessary for plants through the iron carrier it secretes and can deprive harmful microorganisms of the iron required for growth, ultimately facilitating the growth and development of plants [13,14]. Nevertheless, there are few studies dedicated to examining the effects of PGPR on alleviating stress caused by the presence of heavy metals such as Pb, and they primarily focus on Cu, Cd, Ni, and Hg [15].

The bioremediation process encompasses the utilization of various organisms, including bacteria, microalgae, fungi, and plants, to eliminate harmful substances from contaminated environments or to transform these substances into less toxic or innocuous forms [16]. Microorganisms possess the ability to endure environments characterized by high levels of trace metal pollution through mechanisms of detoxification or tolerance. This capability is essential for the processes of species formation, as well as the bioavailability and mobility of trace metals [17]. Numerous investigations have been undertaken to assess the biodegradation capabilities of bacteria or fungi isolated from soil contaminated with metals, specifically in relation to trace metal elements. Fatima Abdullahi Harun et al. [18] reported the isolation and optimization of two novel lead-resistant isolates from an active goldmine-contaminated site of Anka in Zamfara State, Nigeria. The two isolates demonstrated the ability to tolerate lead nitrate concentrations of up to 3000 mg/L. It was observed that mercury, a toxic trace metal, significantly inhibited the growth of both isolates at a significance level of *p* < 0.05. The locally isolated strains of *Paenibacillus* sp. and *Bacillus* sp. show potential as effective agents for the bioremediation of environments contaminated with lead. Additionally, microbial-mediated biogeochemical processes that convert lead into stable precipitates such as phosphate, sulfide, and carbonate are examined within a genetic, metabolic, and systematic framework. This analysis pertains to their application in both laboratory and field settings for the immobilization of environmental lead [19]. Debjani Mandal et al. [20] isolated a plant growth-promoting bacterium exhibiting hypertolerance to trace metals from arsenic-contaminated soil in the Bhagobangola I block of Murshidabad district. The bacterial isolate was classified as belonging to the genus Microbacterium. Within the genome of this secondary oxidizing bacterium, genes and gene clusters associated with arsenic tolerance, as well as tolerance to other trace metals such as copper, manganese, and zinc, were identified alongside those linked to plant growth promotion. The plant growth-promoting rhizobacteria (PGPR) identified in this research may be utilized to enhance plant growth and assist in the microbial remediation of soil contaminated with lead and other trace metals. These microorganisms possess the potential to function as a bioremediation agent and a biological fertilizer, thereby mitigating lead toxicity and fostering plant development.

*Medicago sativa* L. is a perennial flowering plant of the *Medicago* genus in the legume family renowned for its nutritional, economic, and scientific research value as well as its exceptional palatability, earning it the esteemed title of the “King of forage” [21]. Previous studies conducted by our research group have demonstrated that an abundance of rhizosphere microorganisms can enhance the growth of *Trifolium pratense* L. in soils with a lead concentration of 5000 mg/kg [22]. The concentration of lead (Pb) in the soil is approximately 33 times greater than the threshold of 150 mg/kg established by the United States Environmental Protection Agency (EPA). Furthermore, it exceeds the European Union’s Soil Pollution Risk Assessment Guidelines (PRTR) limit of 100 mg/kg by a factor of 50 and is 100 times higher than the Chinese national standard, “Soil Environmental Quality Standards” (GB 15618-2018 [23]), which sets the limit at 50 mg/kg. In light of these findings, the present study seeks to isolate, select, and identify rhizobacteria that promote plant growth and exhibit tolerance to lead, with the objective of enhancing the growth of Medicago sativa L. and developing a strategy for the assisted remediation of lead-contaminated soils. These bacteria were then inoculated onto alfalfa seeds and seedlings under Pb pollution conditions to observe their effects on seed germination and seedling development at varying concentrations of lead pollution. The ultimate goal was to obtain superior lead-resistant strains capable of promoting the growth and development of alfalfa under lead stress, thereby providing essential theoretical support and reference points for plant-microorganism combinations in remediating Pb-contaminated soil.

## 2. Materials and Methods

### 2.1. Experimental Material

The rhizosphere soil of red clover growing in soil with a concentration of 5000 mg/kg was utilized as the experimental material (this rhizosphere soil was stored in a −80 °C refrigerator) [22]. The same variety of alfalfa seed, supernova, was collected from the experimental field of Northeast Agricultural University in Harbin, China (E 126°14′; N 45°05′), and was used for both the germination test and pot experiment. Seed germination tests were conducted in sterile glass Petri dishes. The pots used in the experiment had dimensions of 11.6 × 10.5 cm (inside diameter × height) and contained conventional horticultural soil mixed with vermiculite. Soil lead pollution was simulated by adding lead nitrate solution. After adding the corresponding concentration of lead solution, passivation took place in a dark location for one week, followed by sterilization for 30 min after bagging once passivation was completed. Soil sterilization using HH-4 constant temperature water bath from Guohua Electric Co., Ltd. (Changzhou, China) (program: 121 °C, 40 min).

### 2.2. Isolation and Identification of Lead-Resistant PGPR Strains

Weigh 5 g of the sample rhizosphere soil and place it into a triangular glass bottle containing 45 mL of sterile water. Shake the mixture at 28 °C at 180 rmp for 30 min. At this point, the concentration of bacterial suspension is 10^−1^, followed by five rounds of tenfold gradient dilution to achieve a concentration of 10^−5^. Extract 100 μL of bacterial suspension from each diluent labeled as 10^−3^, 10^−4^, and 10^−5^; add it to LB, R2A, Kogler No. 1 and Beef Extract peptone solid medium center; then use a disposable bacterial inoculation ring to evenly spread the bacterial solution onto the culture medium. (LB medium: 10 g of sodium chloride, 5 g of yeast extract, 10 g of tryptone, 15 g of agar, pH 7.0–7.2, diluted to 1 L with deionized water; R2A medium: 0.5 g of glucose, 0.5 g of starch, 0.5 g of acid-hydrolyzed casein, 0.5 g of bacteriological peptone, 0.5 g of yeast extract, 0.3 g of sodium pyruvate, 0.3 g of hydrogen dihydrogen phosphate, 0.05 g of magnesium sulfate, 2 g of TES, 15 g of agar, pH 7.0, diluted to 1 L with deionized water; Kogler No. 1: 20 g of soluble starch, 1 g of potassium nitrate, 0.5 g of magnesium sulfate, 0.01 g of ferrous sulfate, 0.5 g of dipotassium dihydrogen phosphate, 0.5 g of sodium chloride, 18 g of agar, pH 7.2–7.4, 1 L of deionized water; beef extract peptone solid medium: 5 g of beef extract, 10 g of peptone, 5 g of sodium chloride, 15 g of agar, pH 7.0–7.2, diluted to 1 L with deionized water.) Invert the culture medium in a 28 °C incubator and select a single colony from LB solid medium to coat onto a new solid LB medium until a single purified strain is obtained. Store the purified strains in glycerol at −80 °C. Extract the DNA of the isolated strain using a kit (CWBIO Bacteria Genomic DNA Kit) and perform PCR amplification with universal primers: 27F (5′-AGAGTTTGATCCTGGCTCAG-3′) and1492R (5′-CtacGGCTACCTTgTTACGA-3′). The PCR reaction system is 50 μL. PCR reaction procedure: predenaturation at 95 °C for 5 min, denaturation at 95 °C for 30 s, annealing at 58 °C for 30 s, extension at 72 °C for 90 s, and final extension at 72 °C for 7 min, with a total of 35 cycles. Send the PCR products to Shanghai Piceno Biotechnology Co., Ltd. (Shanghai, China) for bacterial16S rDNA sequencing. Conduct sequence comparison and analysis using BLAST program on NCBI website based on the sequence results, selecting model strains with high homology with 16S rDNA sequence. Construct phylogenetic evolutionary tree using MEGA7.0 software.

### 2.3. Characteristics of Lead-Resistant PGPR Strains

We examined the growth-promoting properties of the target strain, including IAA production capacity, protease production capacity, inorganic phosphorus dissolution capacity, ACC deaminase activity, iron carrier production capacity, and cellulase production capacity. The synthetic ability of IAA was determined in accordance with Thakuria D et al. [24], and the standard curve of IAA is presented in Supplementary Figure S1a. Inoculate the purified target strains in LB liquid medium containing 300 μg/mL L-tryptophan for enrichment and cultivation for 24 h. Take 1 mL of bacterial suspension and add an equal amount of Salkowski color reagent. If the color turns red, it indicates that the strain can produce IAA. The protease production capacity of isolated strains was measured following the method of De Marco J L [25]. Inoculate the activated target strain onto a protease screening medium, invert and culture for 48 h, and observe the outer circle of the colony. If a transparent aperture is produced, it indicates that the strain has protease production ability. The ability to dissolve inorganic phosphorus was evaluated according to Devau N [26]. Inoculate the activated target strains onto inorganic phosphorus medium (NPA), seal the culture dish, invert and culture for one week, and observe the transparent aperture on the outer circle of the colony to indicate that the strain has the ability to dissolve inorganic phosphorus. ACC deaminase activity was determined using a modified method based on work by Penrose and Glick (Penrose and Glick, 2003) [27], with the standard curve of α-butanolic acid content shown in Supplementary Figure S1b. Inoculate the activated target strain onto the ACC deaminase screening medium (ADF), and after 48 h of cultivation, if the bacteria grow normally, it indicates that the strain has ACC deaminase activity. Ferric support production capacity was determined using the chromium azolsulfonate (CAS) analysis method [28]. Cellulase production capacity was assessed with reference to relevant literature [29]. The activated target strains were inoculated onto the Congo red isolation and screening medium and cultured for 3 days. Observe whether a transparent halo forms around the outer edge of the colony. If a transparent halo forms, it indicates that the strain has the ability to produce cellulase. Each experiment was replicated thrice.

### 2.4. Growth Analysis of Strains Under Lead Stress

The plate confrontation method [30] was utilized to confirm the presence of antagonism between the target strains. To observe the morphology and structure of the isolated strains, a single colony screened from the purification plate was activated and added into 50 mL of Lysogenic Broth (LB) medium, oscillated at 28 °C and 200 rmp for 48 h. The bacterial solution was then adjusted to an absorbance value of 0.5 ± 0.02 using sterile water. LB liquid medium containing lead nitrate at concentrations of 0, 250, 500, 1000, and 5000 mg/L was prepared. Subsequently, 1 mL of the target bacterial solution was absorbed and poured into each LB medium containing lead, which was placed in a shaking table at 180 r/min and cultured at 28 °C. The absorbance 600 values were determined every 3 h to draw the growth curve. The physiological and biochemical analysis of these selected target strains was conducted with reference to the Manual for Systematic Identification of Common Bacteria [31].

### 2.5. Pot Experiment

To assess the growth promotion effect of lead-tolerant, growth-promoting strains on alfalfa under varying concentrations of lead stress, the screened lead-tolerant, growth-promoting bacteria and its mixed solution were inoculated into potted alfalfa. Three different levels of lead stress treatments were applied: 0, 1000, and 5000 mg/kg. The pot experiment was conducted at Northeast Agricultural University.

Alfalfa seeds of suitable size and full grain were carefully selected and sterilized using the same method as the germination test. Following sterilization, the seeds were placed in Petri dishes lined with aseptic filter paper. Each dish was then inoculated with 10 mL of target bacteria solution, and the seeds were allowed to soak for 48 h. Subsequently, the soaked seeds were transferred to Petri dishes for germination. After germination, seeds displaying similar growth potential and good performance were chosen and transplanted into POTS containing passivated and sterilized horticultural soil. The substrate utilized for potted plants consists of standard horticultural soil, supplemented with vermiculite. To simulate soil lead contamination, a lead nitrate solution was incorporated. Following the addition of the specified concentration of lead solution, the mixture was allowed to undergo passivation in a dark environment for a duration of one week. Subsequently, the treated soil was packaged and subjected to sterilization for a period of 30 min. Eight alfalfa seeds were sown in each pot. The soil was watered with 100 mL every 3 days to maintain appropriate moisture levels, while a bacterial solution with an absorbance 600 of 0.50 ± 0.02 (1 × 10^6^ CFUmL^−1^) was applied to the plant roots every 5 days (the mixed bacterial solution consisted of a single bacterial suspension with an absorbance 600 of 0.50 ± 0.02 in equal volume), at a dosage of 50 mL each time, in order to sustain stable bacterial abundance in the soil. After 30 days of alfalfa growth, the influences of PGPR applied alone or in combination on the growth of alfalfa under lead stress were investigated.

### 2.6. Plant Sample Analysis

The morphological indexes included in the study are plant height, root length, fresh weight above ground, dry weight above ground, fresh weight of root, and dry weight of root. Plant height refers to the distance from the root neck to the top of the potted plant, while root length indicates the length of the main root of each plant. Aboveground fresh weight represents the combined weight of above ground parts in a group consisting of 5 plants. Similarly, aboveground dry weight denotes the combined weight when sample weights remain to constant weight after drying 5 plants to a constant temperature at 65 °C. Fresh root weight signifies the combined weight of roots from a group with their aboveground parts removed every 5 plants. Lastly, dry root weight represents the combined weight after drying at 105 °C for 30 min when sample weights remain unchanged and then dried to constant temperature at 65 °C every five plants in a group.

Physiological indices include chlorophyll content, malondialdehyde (MDA) content, superoxide dismutase (SOD) activity, peroxidase (POD) activity, and catalase (CAT) activity. The determination of chlorophyll a, chlorophyll b, total chlorophyll, and carotenoids was conducted using the method described by Datt B. [32]. The content of MDA [33] was determined using the thiobarbituric acid (TBA) method. SOD activity was assessed using the nitrogen blue tetrazole (NBT) photo reduction method [34]. POD activity was measured using the guaiacol method, while CAT activity was determined via ultraviolet absorption analysis [35].

The aboveground portion of the plant sample was isolated from the root, dried, and ground, and subsequently, 10 mL of nitric acid was added thereto. It was dissolved into a colorless and transparent solution (180 °C, 90 min) using the microwave digestion system. The lead content in the digestion was determined via xylenol orange spectrophotometry [36].

### 2.7. Soil Sample Analysis

The pH of the soil samples was ascertained using a pH meter with a soil-to-water ratio of 1:1 (*v*:*w*). The samples were agitated using a shaker for 20 min and subsequently determined after a 5 min standing period. The content of bioavailable phosphorus (AP) in 1 g of soil sample was determined via molybdenum-antimony colorimetry (OD700) after 7 mL of ammonium fluoride extract solution was added to the test tube, shaken for 1 min, and filtered. After 5 g of 20-mesh sieved soil was placed in 50 mL of neutral ammonium acetate solution and shaken for 30 min, the content of available potassium (AK) in the filtered filtrate was determined using a flame photometer. After drying and sieving, 0.1 g of soil sample was supplemented with 10 mL of nitric acid and 0.5 mL of hydrofluoric acid, which was digested into a colorless transparent solution using a microwave digestion system (180 °C, 90 min), and the lead content in the digestion was determined via xylenol orange spectrophotometry [32].

### 2.8. Calculation of Biological Concentration Coefficient and Transport Coefficient

Biocon Centration Factor (BCF) and Transfer Factor (TF) can indicate the accumulation of lead in alfalfa and the transfer of lead from the roots to the ground. The calculation formula is as follows:

(1) $$\mathrm{BCF}=\frac{\mathrm{Cplant}}{\mathrm{Csoil}}$$

where Cplant and Csoil are Pb concentrations (mg/kg) in alfalfa and rhizosphere soils, respectively. The Cplant content in alfalfa was calculated as the sum of the Pb contents (mg/kg) in the shoots and roots.

(2) $$\mathrm{TF}=\frac{\mathrm{Cshoot}}{\mathrm{Croot}}$$

where Cshoot and Croot are the Pb concentrations (mg/kg) in aboveground (stem and leaves) and underground (roots) parts of rye grass, respectively [37].

### 2.9. Data Analysis

Excel 2007 was used for data sorting and performing one-way ANOVA and significance analysis using IBM SPSS Statistics 26, with three biological replicates in each group, and the difference significance was defined as *p* < 0.05. Origin 2021 was used for mapping. The images were merged using Adobe Photoshop 2019.

## 3. Results

### 3.1. Preliminary Identification of Lead-Resistant PGPR Strains

In this experiment, 52 species of bacteria isolates with different colony morphologies were initially isolated from the rhizosphere soil of red trefoil artificially contaminated with lead using four types of media: LB, R2A, beef extract peptone, and Kogler No. 1. A total of 20 strains of bacteria with different morphologies were cultured using R2A medium, while 16 strains were cultured using LB medium, 10 strains using beef extract peptone medium, and 6 strains using Kogler No. 1 medium. Additionally, a preliminary qualitative screening was conducted on the 52 bacterial strains to determine their biochemical features typically associated with plant growth promotion. Some positive results of growth-promoting ability identification can be seen in Supplementary Figure S2, and the corresponding results for all 52 bacterial strains are presented in Table S1.

### 3.2. Growth-Promoting Properties of Lead-Resistant PGPR Strains Containing ACC Deaminase

The results indicated significant differences in the growth-promotion characteristics of 52 strains. Y2 and Y10 exhibited IAA production capacities of 27.55 μg/mL and 24.86 μg/mL, respectively, with Y2 showing significantly higher IAA production compared to other bacteria with similar growth-promoting functions (*p* < 0.05). Conversely, Y3 and Y41 demonstrated the weakest IAA production capacities at 2.76 μg/mL and 2.55 μg/mL, respectively (Supplementary Figure S3a). By comparing the D/d values of 26 strains with protease-producing capacity, the strength of their protease-producing capacity could be preliminarily determined. Among them, Y35 and Y2 had higher D/d values, reaching 3.85 and 3.78 respectively, while Y17 and Y6 had smaller D/d values at only 1.73 and 1.59 (Supplementary Figure S3b). Y22, Y25, Y41, and Y45 formed a transparent phosphorus-soluble circle around their colonies, indicating their ability to dissolve calcium phosphate. The results showed that compared with the other three strains, Y22 had the highest D/d value (2.42). The D/d values of the other three strains were similar, with Y45 being the smallest (1.87) (Supplementary Figure S3c). As illustrated in (Supplementary Figure S3d), the ACC deaminase activity of strain Y21 was the highest, at 0.5523 U/mg, which was significantly greater than that of other strains (*p* < 0.05). The ACC activity of strains Y22, Y46, and Y50 did not show significant differences, while the ACC activity of strains Y24 and Y45 was the lowest and significantly lower than that of other strains (*p* < 0.05). Strains Y23, Y27, and Y41 were all capable of secreting ferriophore. Among them, strain Y23 exhibited the largest D/d value, at 2.01; strain Y27 had a D/d value of 2, similar to that of strain Y23; and strainY41 had the smallest D/d value, at 1.71 (Supplementary Figure S3e). Among the 6 strains exhibiting cellulase activity, Y32 demonstrated the highest D/d value (2.43), followed by Y31. Both Y23 and Y33 had the same D/d value of 2.30, while Y52 exhibited the lowest D/d value of 1.73 (Supplementary Figure S3f). In conclusion, it was found that the growth-promoting ability of Y2, Y22, and Y23 was significantly higher than that of other strains (*p* < 0.05) (Table 1).

### 3.3. Morphological Characteristics of Lead-Resistant PGPR Strains

**Figure 1:**
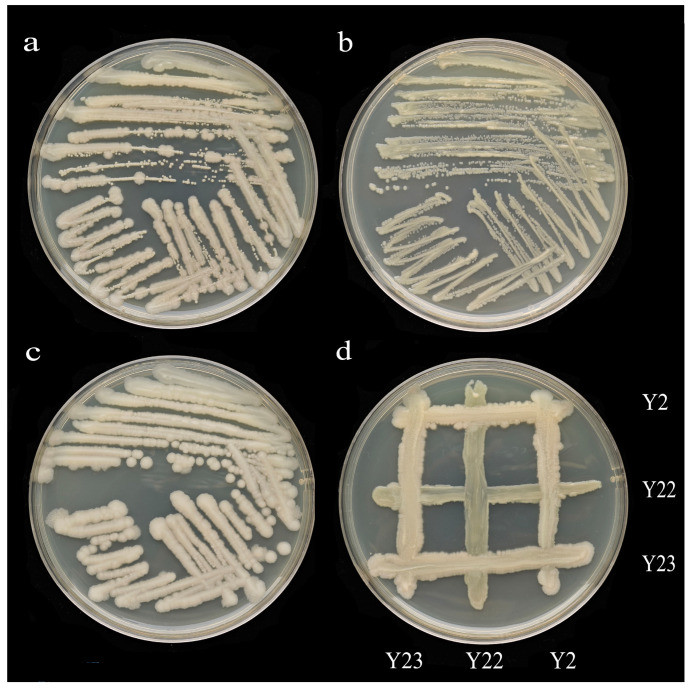
Characteristics of tested strains. (**a**–**c**) Strain morphology ((**a**) was Y2, (**b**) was Y22, and (**c**) was Y23). (**d**) Strain antagonism test. The agar plate confrontation method was employed to inoculate the selected target strains into a uniform LB solid culture medium. This was accomplished using a sterilized inoculation ring and a cross-shaped streaking technique. Subsequently, the plates were inverted and incubated at a constant temperature of 28 °C for a duration of 48 h. The interaction zone between the bacterial strains was examined to determine the presence of a transparent zone. The absence of such a zone suggested that the target strains could coexist, whereas the presence of a transparent zone indicated an antagonistic interaction between the strains, precluding their coexistence.

By comparing the growth-promotion ability of the strains, Y2, Y22, and Y23 strains were selected for further studies. The morphology of the tested strains is depicted in (Figure 1a–c). The morphology of the three strains differed: the Y2 colony exhibited a slight bulge with a milky white color and rough surface, along with folds inside; the Y22 colony was nearly round, with a white color, smooth surface, and flocculent interior; while the Y23 colony had a smooth surface, milky color, and bulge. The results of antagonism tests indicated that there was no antagonism between these three strains, as they could coexist (Figure 1d). Their respective growth-promoting functions are presented in Table S2. The microscopic morphology of these strains was observed using scanning electron microscopy, as shown in Supplementary Figure S4.

### 3.4. 16Sr DNA Sequence Analysis of Lead-Resistant PGPR Strains

**Figure 2:**
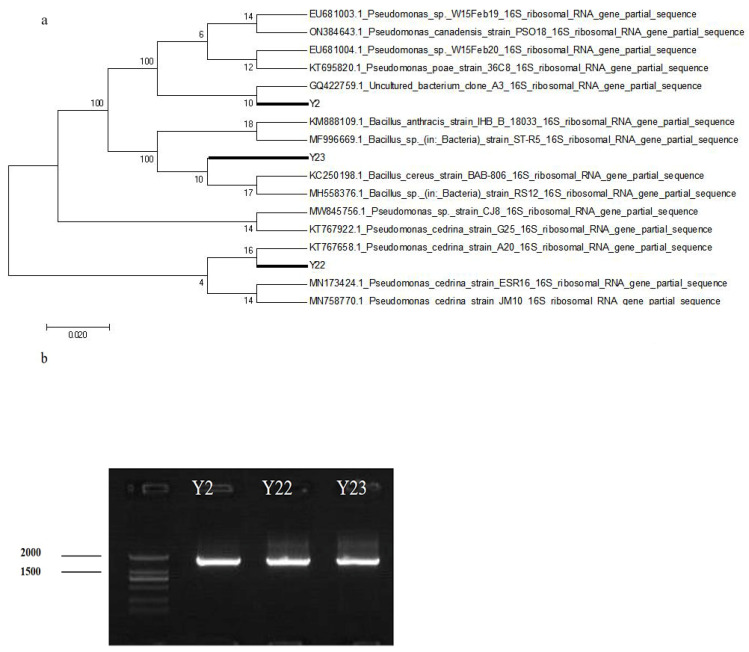
Sequencing results of the tested strains. (**a**) Electrophoretic map of PCR products of test strain. (**b**) Phylogenetic tree of test strain.

The DNA of the three strains was extracted, and PCR amplification was performed using 27F and 1492R universal primers to obtain three fragments with sizes of 1441 bp for Y2, 1405 bp for Y22, and 1464 bp for Y23, as shown in (Figure 2a). PCR products were obtained after the purification of each strain. Subsequently, DNA sequencing was carried out using the ABI3730-XL sequencing instrument. Sequence comparison and analysis were conducted using the BLAST program on the NCBI website based on the sequencing results. A phylogenetic tree was constructed using the maximum likelihood method, as shown in Figure 2b. According to the identification results, strains Y2 and Y22 belong to the genus *Pseudomonas*, while strain Y23 belongs to the genus *Bacillus.*

### 3.5. Effects of Lead Stress on the Growth of PGPR Strains

**Figure 3:**
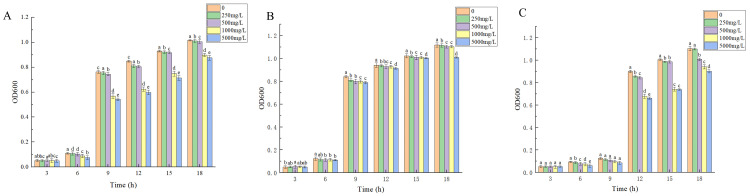
Growth of experimental strains under different lead concentration stress at different stress times. (**A**) Y2. (**B**) Y22. (**C**) Y23. LB medium: 10 g of sodium chloride; 5 g of yeast extract; 10 g of tryptone; 15 g of agar; pH 7.0–7.2; 1 L of deionized water; and 0, 250, 500, 1000, and 5000 mg/L of lead nitrate. Three biological replicates were conducted for each experiment. Different lowercase letters indicate significant differences at different lead treatment concentrations at the same time (*p* < 0.05).

The growth curves of three tested strains under lead stress are depicted in Figure 3. It was observed that all three strains of bacteria were able to grow under lead stress, with the higher concentration of lead stress (>1000 mg/L) having a greater impact on Y2 and Y23. However, it could not completely inhibit their growth. On the other hand, Y22 was less affected by lead stress, and there was no significant change in its growth trend under different concentrations of lead stress.

### 3.6. Effect of Lead-Resistant PGPR Strain Containing ACC Deaminase on Growth of Alfalfa Seedlings

**Figure 4:**
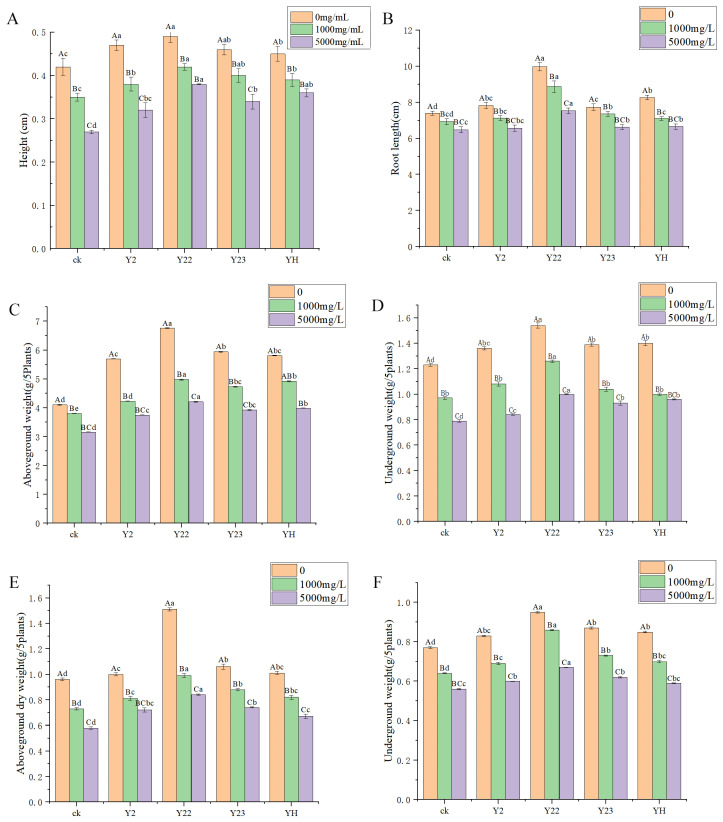
Effect of strains on alfalfa seedlings under lead stress. (**A**) Effects of strains on plant height of alfalfa under different concentrations of lead stress. (**B**) Effects of strains on root length of alfalfa under different concentrations of lead. (**C**) Effects of strains on fresh weight of alfalfa under different concentrations of lead. (**D**) Effects of strains on fresh weight of underground alfalfa under different concentrations of lead. (**E**) Effects of strains on dry weight of aboveground alfalfa under different concentrations of lead stress. (**F**) Effects of strains on underground dry weight of alfalfa under different concentrations of lead stress. Three biological replicates were conducted for each experiment. Different capital letters indicate significant differences in the same treatment under different lead concentrations. Different lowercase letters indicate significant differences (*p* < 0.05) between different treatments at the same lead concentration.

Alfalfa seedlings were subjected to different concentrations of lead in Y2, Y22, and Y23 bacterial solution and mixed bacterial solution. After 30 days, physiological and biochemical indexes, such as plant height, root length, fresh weight, and dry weight, were measured (Figure 4). The results indicated that under both low-lead and high-lead treatments, the plant height (Figure 4A) and root length (Figure 4B) of alfalfa significantly increased with Y22 compared to the control group. Additionally, the fresh weight of the aboveground part (Figure 4C) and underground part (Figure 4D) was highest with the Y22 treatment. Furthermore, the dry weight of the aboveground part (Figure 4E) and underground part (Figure 4F) also showed significant increases with the Y22 treatment compared to the control group. Y2 significantly increased the plant height, root length, aboveground fresh weight, and aboveground dry weight of alfalfa. It also decreased the underground fresh weight and underground dry weight of the plant. Similarly, Y23 and YH (a mixture of Y2, Y22, and Y23) had a significant impact on increasing plant height, root length, aboveground fresh weight, and aboveground dry weight while reducing underground fresh weight and underground dry weight of alfalfa under low-lead-concentration treatment.

### 3.7. Effect of Lead-Resistant PGPR Strain on Antioxidant Enzyme Activity of Alfalfa Seedlings

**Figure 5:**
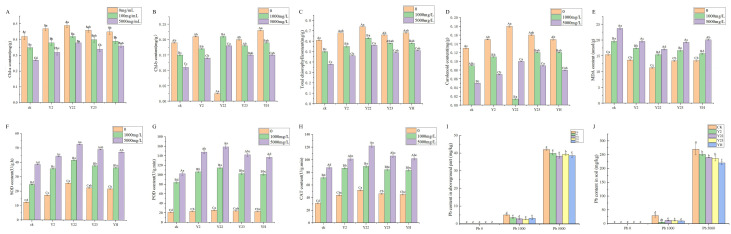
Effects of strains on chlorophyll and antioxidant properties of alfalfa seedlings under lead stress. (**A**) chlorophyll a, (**B**) chlorophyll b, (**C**) total chlorophyll, (**D**) carotenoids, (**E**) MDA, (**F**) SOD, (**G**) POD, and (**H**) CAT. (**A**–**H**) Different capital letters indicate significant differences in the same treatment under different lead concentrations. Different lowercase letters indicate significant differences (*p* < 0.05) between different treatments at the same lead concentration. (**I**) Lead content in ground parts under different Pb stress. (**J**) Lead content in underground parts under different Pb stress. Three biological replicates were conducted for each experiment. (**I**,**J**) Different lowercase letters indicate that under different concentrations of lead stress, the lead content in soil treated with different bacterial solutions is significantly reduced compared to CK (*p* < 0.05).

As depicted in (Figure 5), inoculation led to an increase in the levels of chlorophyll a, b, total chlorophyll, and carotenoid in alfalfa while decreasing MDA, SOD, POD, and CAT. With the rise in the lead concentration, there was a significant decrease (*p* < 0.05) in the levels of chlorophyll a, b, total chlorophyll, and carotenoid across all treatment groups. Additionally, compared to alfalfa without lead stress, MDA, SOD, POD, and CAT levels were significantly increased (*p* < 0.05) in alfalfa treated with Y2, Y22, Y23, YH, and CK.

Furthermore, it was observed that under high-lead-stress conditions, the malondialdehyde content in alfalfa inoculated with Y22 was notably reduced by 27.85% compared to CK (Figure 5E), while the activities of SOD (Figure 5F), POD (Figure 5G), and CAT (Figure 5H) were significantly higher than those treated with CK (*p* < 0.05). Lead contents in aboveground and underground parts of alfalfa under different concentrations of lead stress are shown in (Figure 5I,J). Compared with the CK group, PGPR treatment significantly reduced lead contents in aboveground and underground parts of alfalfa, among which the YH mixed bacterial solution group significantly reduced lead contents.

### 3.8. Effects of Lead-Resistant PGPR Strains on Physicochemical Properties of Soil Samples

**Figure 6:**
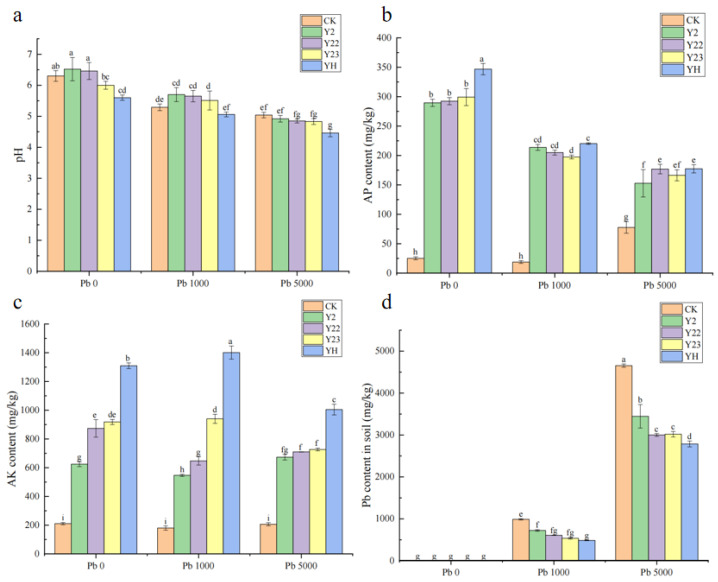
Contents of (**a**) pH, (**b**) AP, (**c**) AK, and (**d**) Pb in rhizosphere soil samples of alfalfa under different treatments. Different lowercase letters indicate significant differences in the properties of soil treated with different bacterial solutions under different lead stress concentrations compared to CK (*p* < 0.05).

The effect of PGPR on soil pH under diverse lead concentration pollution is depicted in Figure 6a. When PGPR is not inoculated in the presence of lead pollution, the soil pH value is 6.3, and the soil pH value decreases as the lead pollution concentration rises. In the Pb5000 treatment, the soil pH value attains the minimum value, which is 29.21% lower than that of Pb0. PGPR treatment augmented the contents of P and K in the rhizosphere soil of alfalfa, and the data of different groups exhibited significant differences, indicating that distinct treatments had dissimilar effects on the contents of AP and AK in the soil in the experiment. As can be observed in Figure 6b,c, the simultaneous inoculation of three types of bacteria is more efficacious than a single strain in resolving P and K. Compared with the CK group, PGPR treatment significantly decreased the Pb content in the rhizosphere soil, among which the SKP group significantly reduced the Pb content (Figure 6d). Compared to the control, PGPR enhanced the BCF and TF values of alfalfa against Pb (Table 2).

## 4. Discussion

Phytoremediation has emerged as a promising method for environmental pollution remediation in recent years. It offers the advantages of being cost-effective, environmentally friendly, and more readily accepted by the public [38]. Soil microorganisms, particularly rhizosphere growth-promoting bacteria, play a crucial role in soil development, fertility formation, nutrient cycling, plant growth, and disease control [39]. Research has demonstrated that these bacteria can enhance plant growth under stress conditions and promote plant recovery through direct and indirect mechanisms. Among these mechanisms, rhizosphere growth-promoting bacteria primarily stimulate plant growth by solubilizing phosphorus, secreting growth-regulating substances (such as IAA and ACC deaminase), and producing iron carriers [40]. Screening for suitable rhizosphere growth-promoting bacteria can be conducted based on these mechanisms [41]. In this study, three outstanding PGPR strains were selected from lead-resistant red trilobium rhizosphere soil. Specifically, strain Y2 was found to produce auxin and secrete protease; strain Y22 exhibited the functions of producing auxin, secreting protease, decomposing inorganic phosphorus, and producing ACC deaminase; while strain Y23 was identified as capable of secreting protease, promoting ferriferite production, and synthesizing cellulose enzymes.

As a crucial class of hormone substances that affect plant growth, IAA plays a significant role in plant growth and development by promoting cell division [42]. Additionally, IAA has the ability to stimulate the elongation of plant roots and modify their nutrient and water absorption patterns, thereby facilitating plant growth in trace metals soil [43]. Malik et al. [44] employed the colorimetric approach to assess the IAA secretion capacity of rhizosphere nitrogen-fixing strains of wheat and other plants in the saline-alkali soil of Pakistan, which ranged from 5.11 to 35.70 mg/L. Among the three strains screened in this research, Y2 and Y22 exhibited growth and production capabilities. The auxin produced by Y2 was significantly higher than that of other strains in terms of auxin-producing ability, and the content of IAA produced was 27.55 μg/mL when the L-Trp concentration was 300 μg/mL.

Studies have demonstrated that plant endophytic bacteria harboring ACC deaminase exert a significant role in facilitating plant growth and enhancing plant stress tolerance [45]. Both endophytes, P. brassicacearum YsS6 and P. migulae 8R6, manifested ACC deaminase activity, which not only stimulated the growth of tomato plants under non-stress circumstances but also ameliorated the growth of tomato plants under salt stress in contrast to unvaccinated plants or plants inoculated with the corresponding Pseudomonas acdS−mutant. Tomato plants were induced to have a higher accumulation of fresh and dry biomass, a greater chlorophyll content, and a larger number of flowers and buds [46]. Ashrafuzzaman’s study suggested that the increase in rice seedling growth following the introduction of PGPR may be attributed to the stimulation of IAA production and phosphorus dissolution, indicating that the plant growth-promoting ability of this strain is associated with other growth-promoting characteristics [47]. The Y22 strain identified in this study exhibited a high yield of ACC deaminase, inorganic phosphorus dissolution ability, and a D/d value of 2.42, signifying its significant research value.

Soil protease plays a crucial role in evaluating soil health and establishing sustainable farming practices [48]. Lead stress can impact soil enzyme activity induced by microorganisms. Research conducted by Qi R et al. demonstrated that the increase in soil protease activity was accompanied by a rising trend in soil organic carbon content [49]. Furthermore, soil microbial communities can influence the decomposition of soil organic matter through various soil enzymes [50]. All three strains screened in this study exhibited the ability to produce protease. Treatment with the bacterial solution of these three strains, as well as their mixed bacterial solution, may enhance the organic matter and DOM content in the soil by increasing protease activity, thereby improving alfalfa growth in lead-stressed soil.

pH serves as an indicator of proton effectiveness in trace metal-contaminated soils, thereby indirectly reflecting alterations in soil conditions across varying pollution levels [51]. Plant Growth-Promoting Rhizobacteria (PGPR) secrete low-molecular-weight organic acids that enhance the mobility of metal ions within the soil and increase both their bioavailability and adsorption by root systems [52]. In this study, analogous trends were observed regarding pH changes. At elevated lead concentrations (>5000 mg/L), the pH value in the YH group was significantly lower than that of the control group; furthermore, after treatment with three different lead concentrations, all values for the YH group remained reduced, suggesting that simultaneous inoculation with three strains exerted a pronounced effect on pH. Research indicates that PGPR can biologically mitigate soil contamination when subjected to pollutants [53], potentially due to Pb^2+^ infiltration into plant tissues, which alters physiological behaviors—particularly enhancing enzymatic activity—though further investigation is warranted to elucidate these mechanisms [54]. Rasool’s study also demonstrated that applying rhizospheric growth-promoting bacteria could enhance sunflower growth under lead stress, increase its yield and antioxidant activity, elevate proline content, and reduce damage to sunflower somatic cells [55]. Ali Q et al. investigated the seedling stage of rice (*Oryza sativa* L.) under Cd stress and discovered that the supplementation of rhizosphere growth-promoting bacterial solution and bactericide could enhance the growth of rice under Cd stress and mitigate the negative impacts of Cd on rice [56]. In this investigation, it was observed that under low lead stress, the treatment of 3 strains of bacteria and a mixed bacterial solution significantly increased the plant height of alfalfa. Additionally, the fresh dry weight of the underground part of alfalfa was notably enhanced by the Y22 bacterial solution treatment, which may be attributed to the IAA and ACC deaminase production ability of strain Y22. Under high-lead treatment, both plant height and aboveground fresh dry weight of alfalfa were significantly increased by treatments involving three strains of bacteria and a mixed bacterial solution. It is evident that rhizosphere growth-promoting bacteria demonstrate their capacity for promoting plant growth in such conditions, particularly through their production of IAA and ACC deaminase.

The chlorophyll content serves as an indicator of the photosynthesis and nutritional status of plants [57]. Heavy metals have been found to significantly decrease the chlorophyll content in plants, as they disrupt the formation of chlorophyll precursor substances, leading to structural damage and reduced photosynthetic efficiency [58,59]. In this study, as the lead concentration increased, the chlorophyll content in alfalfa demonstrated a significant downward trend. However, upon the addition of three strains of bacteria and the mixed bacterial solution, the chlorophyll content in alfalfa was conspicuously enhanced. This aligns with the discoveries of Montes-Osuna et al. [60], who observed that the supplementation of rhizosphere growth-promoting bacteria could augment the chlorophyll content in olive trees (*Olea europaea* L.), suggesting that the addition of PGPR ameliorates the nutrition and growth of alfalfa under lead stress. This might result from the preference of PGPR for the occupation of the plant rhizosphere and the facilitation of nutrient absorption by plants. Specifically, it can generate IAA and ACC deaminase and regulate the ethylene content in plants, thereby increasing the chlorophyll content in plants and delaying their aging [61].

Malondialdehyde (MDA) is a product of membrane lipid peroxidation, and it directly reflects the degree of cell membrane damage [62,63]. In this study, the malondialdehyde content in alfalfa plants significantly increased with the increase in the lead concentration. This is because the presence of trace metals induces other microorganisms in plant roots or soil to produce oxygen free radicals, which accelerates plant aging [64]. Under the same level of lead stress, the treatment with three strains of bacteria and a mixed bacterial solution significantly reduced the malondialdehyde content in alfalfa. This indicates that the addition of PGPR significantly alleviated cell membrane damage in alfalfa under lead stress and improved plant tolerance. Additionally, studies have shown that lipopolysaccharide, a main component of Gram-negative bacteria’s outer membrane, helps cells resist external pressure by promoting antioxidant enzymes and ROS production [64,65,66]. Stefanie R et al. [64] demonstrated that lipopolysaccharides purified from the pathogenic bacterium *Pseudomonas aeruginosa* are capable of triggering an innate immune response in Arabidopsis thaliana and inducing a biphasic burst of its reactive oxygen species. Both Y2 and Y22 are pseudomonas and Gram-negative bacteria; therefore, lipopolysaccharides in their outer membranes may have triggered a stress response in alfalfa to some extent. This could result in increased activity of antioxidant enzymes in vivo; however, further research is needed to understand this specific mechanism.

HMs-resistant PGPR can enhance the physicochemical properties of soil and the bioavailability of metals, thereby facilitating plant growth and the rapid removal or transformation of HMs from contaminated soil [67]. In this study, the content of available phosphorus (AP) and available potassium (AK) in soil significantly increased after inoculation with PGPR. The application of PGPR enhanced the content of soil nutrients, promoted plant growth, and resulted in an increase in plants’ utilization of soil elements. Badr N et al. [68] concluded that the phytoremediation effect of soil heavy metal pollution is closely related to the growth status of plants. Compared with other metals, lead is more prone to be complexed or combined with other substances in the soil and thus retained in the soil. In this study, as the lead pollution concentration increased, the lead content in the aboveground and underground parts of alfalfa also rose, indicating that the three PGPRs promoted the migration of Pb from soil to ryegrass under lead stress.

## 5. Conclusions

The results of the potted plant experiment show that inoculating with PGPR can effectively adsorb trace metals in the soil. Under high-lead-concentration treatment, it significantly reduces the lead content in both aboveground and underground parts of purple clover. Compared to the control group without PGPR inoculation, PGPR effectively promotes plant growth and enhances purple clover’s tolerance to lead stress. *Pseudomonas* spp. strain Y22 has the capabilities of generating auxin, secreting protease, decomposing inorganic phosphorus, and producing ACC deaminase. Among different concentrations of lead stress, the YH treatment had the most prominent effect on promoting the growth of alfalfa and remedying trace metals pollution in the soil, which has great potential in the field of joint remediation of trace metals polluted soil with plants. Hence, it is of considerable significance to study the growth-promoting properties of PGPR and its synergetic effect on promoting plant production and regulating the circulation of materials and the flow of energy in the soil.

## Figures and Tables

**Table 1:** The content of auxin and ACC deaminase produced by each strain.

Strain Number	Auxin Content (μg/mL)	ACC Deaminase Content (U/mg)
Y2	27.55 ± 0.12 a	—
Y10	24.86 ± 0.13 b	—
Y12	21.06 ± 0.83 d	—
Y13	18.02 ± 0.13 f	—
Y19	20.86 ± 0.21 d	—
Y22	4.80 ± 0.10 g	0.29 ± 0.02 c
Y24	—	0.19 ± 0.00 d
Y25	18.88 ± 0.49 e	0.45 ± 0.00 a
Y39	21.61 ± 0.11 c	—
Y46	—	0.31 ± 0.02 b
Y50	—	0.29 ± 0.01 c

The values are mean ± standard deviation, and lowercase letters indicate significant differences (*p* < 0.05) in the secretion of auxin and ACC deaminase among different microorganisms.

**Table 2:** BCF and CF of Pb in alfalfa treated with different bacterial solutions under three different concentrations of lead stress.

	Parameters	CK	Y2	Y22	Y23	YH
Pb0	BCF	0	0	0	0	0
	TF	0	0	0	0	0
Pb1000	BCF	0.0250	0.0249	0.0245	0.0250	0.0266
	TF	0.2468	0.2322	0.2306	0.2434	0.3145
Pb5000	BCF	0.0669	0.0843	0.0926	0.0193	0.0928
	TF	0.1559	0.1588	0.1599	0.1664	0.1752

BCF: Biocon Centration Factor, TF: Transfer Factor.

## Data Availability

The original contributions presented in the study are included in the article and Supplementary Materials; further inquiries can be directed to the corresponding author.

## Supplementary Materials

The following supporting information can be downloaded at: https://www.mdpi.com/article/10.3390/microorganisms13010210/s1.

## References

[1] Zhang L., Zhu Y., Gu H., Lam S.S., Chen X., Sonne C., Peng W. (2024). A review of phytoremediation of environmental lead (pb) contamination. Chemosphere.

[2] Yildiz U., Ozkul C. (2024). Heavy metals contamination and ecological risks in agricultural soils of Uşak, western Türkiye: A geostatistical and multivariate analysis. Environ. Geochem. Health.

[3] Sansinenea E. (2019). Bacillus spp.: As Plant Growth-Promoting Bacteria. Secondary Metabolites of Plant Growth Promoting Rhizomicroorganisms: Discovery and Applications, Singh, H.B., Keswani, C., Reddy, M.S., Sansinenea, E., García-Estrada, C., Eds..

[4] Pourrut B., Shahid M., Dumat C., Winterton P., Pinelli E. (2011). Lead Uptake, Toxicity, and Detoxification in Plants. Reviews of Environmental Contamination and Toxicology, Whitacre, D.M., Ed..

[5] Wang F., Jin F., Lin X., Jia F., Song K., Liang J., Zhang J., Zhang J. (2024). Priestia aryabhattai Improves Soil Environment and Promotes Alfalfa Growth by Enhancing Rhizosphere Microbial Carbon Sequestration Capacity Under Greenhouse Conditions. Curr. Microbiol..

[6] Patel S., Jinal H.N., Amaresan N. (2017). Isolation and characterization of drought resistance bacteria for plant growth promoting properties and their effect on chilli (*Capsicum annuum*) seedling under salt stress. Biocatal. Agric. Biotechnol..

[7] Etesami H., Beattie G.A. (2018). Mining Halophytes for Plant Growth-Promoting Halotolerant Bacteria to Enhance the Salinity Tolerance of Non-halophytic Crops. Front. Microbiol..

[8] Bukhat S., Imran A., Javaid S., Shahid M., Majeed A., Naqqash T. (2020). Communication of plants with microbial world: Exploring the regulatory networks for PGPR mediated defense signaling. Microbiol. Res..

[9] Gupta G., Parihar S., Ahirwar N., Snehi D.S.K., Singh V. (2015). Plant Growth Promoting Rhizobacteria (PGPR): Current and Future Prospects for Development of Sustainable Agriculture. Microb. Biochem. Technol..

[10] Etesami H., Glick B.R. (2020). Halotolerant plant growth–promoting bacteria: Prospects for alleviating salinity stress in plants. Environ. Exp. Bot..

[11] Mirzaei M., Moghadam A.L., Hakimi L., Danaee E. (2020). Plant growth promoting rhizobacteria (PGPR) improve plant growth, antioxidant capacity, and essential oil properties of lemongrass (*Cymbopogon citratus*) under water stress. Iran. J. Plant Physiol..

[12] Perruzza L., Zagaglia C., Vitiello L., Sarshar M., Strati F., Pasqua M., Grassi F., Nicoletti M., Palamara Anna T., Ambrosi C. (2023). The Shigella flexneri virulence factor apyrase is released inside eukaryotic cells to hijack host cell fate. Microbiol. Spectr..

[13] Crowley D.E., Wang Y.C., Reid C.P.P., Szaniszlo P.J., Chen Y., Hadar Y. (1991). Mechanisms of iron acquisition from siderophores by microorganisms and plants. Proceedings of the Iron Nutrition and Interactions in Plants: “Proceedings of the Fifth International Symposium on Iron Nutrition and Interactions in Plants”.

[14] Kashyap B.K., Solanki M.K., Pandey A.K., Prabha S., Kumari B. (2019). Bacillus as Plant Growth Promoting Rhizobacteria (PGPR): A Promising Green Agriculture Technology. Plant Health Under Biotic Stress.

[15] Bhanse P., Kumar M., Singh L., Awasthi M.K., Qureshi A. (2022). Role of plant growth-promoting rhizobacteria in boosting the phytoremediation of stressed soils: Opportunities, challenges, and prospects. Chemosphere.

[16] Meyer L., Guyot S., Chalot M., Capelli N. (2023). The potential of microorganisms as biomonitoring and bioremediation tools for mercury-contaminated soils. Ecotoxicol. Environ. Saf..

[17] Ding C., Ding Z., Liu Q., Liu W., Chai L. (2024). Advances in mechanism for the microbial transformation of heavy metals: Implications for bioremediation strategies. Chem. Commun..

[18] Harun F.A., Yakasai H.M., Jagaba A.H., Usman S., Umar H.A., Shukor M.Y. (2024). Bioremediation potential of *Bacillus* sp. and *Paenebacillus* sp. novel lead-resistant isolates: Identification, characterization, and optimization studies. Microbe.

[19] Elizabeth George S., Wan Y. (2023). Microbial functionalities and immobilization of environmental lead: Biogeochemical and molecular mechanisms and implications for bioremediation. J. Hazard. Mater..

[20] Mandal D., Das S.K., Adhikari J., Chatterjee D., Bandyopadhyay T.K., Basu A. (2024). Genome sequencing, annotation and application of a strain of Microbacterium paraoxydans—A bacterium with arsenic bioremediation and plant growth promoting potential. Microbe.

[21] Wang B., Liu Q., Xu W., Yuan Y., Tuluhong M., Yu J., Cui G. (2024). Genome-Wide Identification of MsICE Gene Family in Medicago sativa and Expression Analysis of the Response to Abiotic Stress. Agronomy.

[22] Meng L., Wu Y., Mu M., Wang Z., Chen Z., Wang L., Ma Z., Cui G., Yin X. (2023). Effects of different concentrations of biochar amendments and Pb toxicity on rhizosphere soil characteristics and bacterial community of red clover (*Trifolium pretense* L.). Front. Plant Sci..

[23] (2018). Soil Environment Quality Risk Control Standard for Soil Contamination of Agriculture Land.

[24] Thakuria D., Talukdar N.C., Goswami C., Hazarika S., Boro R.C., Khan M.R. (2004). Characterization and screening of bacteria from rhizosphere of rice grown in acidic soils of Assam. Curr. Sci..

[25] De Marco J.L., Felix C.R. (2002). Characterization of a protease produced by a Trichoderma harzianum isolate which controls cocoa plant witches’ broom disease. BMC Biochem..

[26] Devau N., Hinsinger P., Cadre E.L., Colomb B., Gérard F. (2011). Fertilization and pH effects on processes and mechanisms controlling dissolved inorganic phosphorus in soils. Geochim. Cosmochim. Acta.

[27] Penrose D.M., Glick B.R. (2003). Methods for isolating and characterizing ACC deaminase-containing plant growth-promoting rhizobacteria. Physiol. Plant..

[28] Ferreira C., Boas A.V., Sousa C.A., Soares H.M., Soares E.V.J.A.E. (2019). Comparison of five bacterial strains producing siderophores with ability to chelate iron under alkaline conditions. AMB Express.

[29] Bhadrecha P., Bala M., Khasa Y.P., Arshi A., Singh J., Kumar M. (2020). Hippophae rhamnoides L. rhizobacteria exhibit diversified cellulase and pectinase activities. Physiol. Mol. Biol. Plants.

[30] Cui Y., Zhu Y., Dong G., Li Y., Xu J., Cheng Z., Li L., Gong G., Yu X. (2024). Evaluation of the control efficacy of antagonistic bacteria from V-Ti magnetite mine tailings on kiwifruit brown spots in pot and field experiments. Front. Microbiol..

[31] Schillaci M., Raio A., Sillo F., Zampieri E., Mahmood S., Anjum M., Khalid A., Centritto M. (2022). Pseudomonas and Curtobacterium Strains from Olive Rhizosphere Characterized and Evaluated for Plant Growth Promoting Traits. Plants.

[32] Datt B. (1998). Remote Sensing of Chlorophyll a, Chlorophyll b, Chlorophyll a+b, and Total Carotenoid Content in Eucalyptus Leaves. Remote Sens. Environ..

[33] Draper H.H., Squires E.J., Mahmoodi H., Wu J., Agarwal S., Hadley M. (1993). A comparative evaluation of thiobarbituric acid methods for the determination of malondialdehyde in biological materials. Free Radic. Biol. Med..

[34] Li Z.-X., Lan J.-B., Liu Y.-Q., Qi L.-W., Tang J.-M. (2020). Investigation of the role of AcTPR2 in kiwifruit and its response to Botrytis cinerea infection. BMC Plant Biol..

[35] Wang J., Zhang H., Zhang T., Zhang R., Liu R., Chen Y. (2015). Molecular mechanism on cadmium-induced activity changes of catalase and superoxide dismutase. Int. J. Biol. Macromol..

[36] Du H.Y., Zhang Z.Q., Zhang Y.N., Li M.Q. (2009). Study on colour reaction of xylenol orange with Pb(II) complex and its application. J. Baoji Univ. Arts Sci. (Nat. Sci.).

[37] Zhao Y., Yao J., Li H., Sunahara G., Li M., Tang C., Duran R., Ma B., Liu H., Feng L. (2024). Effects of three plant growth-promoting bacterial symbiosis with ryegrass for remediation of Cd, Pb, and Zn soil in a mining area. J. Environ. Manag..

[38] Yang Y., Xiao C., Wang F., Peng L., Zeng Q., Luo S. (2022). Assessment of the potential for phytoremediation of cadmium polluted soils by various crop rotation patterns based on the annual input and output fluxes. J. Hazard. Mater..

[39] Sindhu S., Singh P., Phour M., Kumari K. (2014). Rhizosphrere microorganisms for improvement in soil fertility and plant growth. Microbes in the Service of Mankind: Tiny Bugs with Huge Impact.

[40] Azizoglu U., Yilmaz N., Simsek O., Ibal J.C., Shin J.H. (2021). The fate of plant growth-promoting rhizobacteria in soilless agriculture: Future perspectives. 3 Biotech.

[41] Shi Z., Guo X., Lei Z., Wang Y., Yang Z., Niu J., Liang J. (2023). Screening of high-efficiency nitrogen-fixing bacteria from the traditional Chinese medicine plant Astragalus mongolicus and its effect on plant growth promotion and bacterial communities in the rhizosphere. BMC Microbiol..

[42] Ortíz-Castro R., Contreras-Cornejo H.A., Macías-Rodríguez L., López-Bucio J. (2009). The role of microbial signals in plant growth and development. Plant Signal. Behav..

[43] Tak H.I., Ahmad F., Babalola O.O. (2013). Advances in the Application of Plant Growth-Promoting Rhizobacteria in Phytoremediation of Heavy Metals. Reviews of Environmental Contamination and Toxicology, Whitacre, D.M., Ed..

[44] Malik K.A., Bilal R., Mehnaz S., Rasul G., Mirza M.S., Ali S. (1997). Association of nitrogen-fixing, plant-growth-promoting rhizobacteria (PGPR) with kallar grass and rice. Opportunities for Biological Nitrogen Fixation in Rice and Other Non-Legumes: Papers Presented at the Second Working Group Meeting of the Frontier Project on Nitrogen Fixation in Rice Held at the National Institute for Biotechnology and Genetic Engineering (NIBGE), Faisalabad, Pakistan, 13–15 October 1996.

[45] Afridi M.S., Mahmood T., Salam A., Mukhtar T., Mehmood S., Ali J., Khatoon Z., Bibi M., Javed M.T., Sultan T. (2019). Induction of tolerance to salinity in wheat genotypes by plant growth promoting endophytes: Involvement of ACC deaminase and antioxidant enzymes. Plant Physiol. Biochem..

[46] Ali S., Charles T.C., Glick B.R. (2014). Amelioration of high salinity stress damage by plant growth-promoting bacterial endophytes that contain ACC deaminase. Plant Physiol. Biochem..

[47] Ashrafuzzaman M., Hossen F.A., Ismail M.R., Hoque A., Islam M.Z., Shahidullah S.M., Meon S. (2009). Efficiency of plant growth-promoting rhizobacteria (PGPR) for the enhancement of rice growth. Afr. J. Biotechnol..

[48] Karaca A., Cetin S.C., Turgay O.C., Kizilkaya R. (2011). Soil Enzymes as Indication of Soil Quality. Soil Enzymology, Shukla, G., Varma, A., Eds..

[49] Qi R., Li J., Lin Z., Li Z., Li Y., Yang X., Zhang J., Zhao B. (2016). Temperature effects on soil organic carbon, soil labile organic carbon fractions, and soil enzyme activities under long-term fertilization regimes. Appl. Soil Ecol..

[50] Puissant J., Cécillon L., Mills R.T.E., Robroek B.J.M., Gavazov K., De Danieli S., Spiegelberger T., Buttler A., Brun J.J. (2015). Biochemistry. Seasonal influence of climate manipulation on microbial community structure and function in mountain soils. Soil Biol. Biochem..

[51] Deng B., Yang K., Zhang Y., Li Z. (2016). Can heavy metal pollution defend seed germination against heat stress? Effect of heavy metals (Cu^2+^, Cd^2+^ and Hg^2+^) on maize seed germination under high temperature. Environ. Pollut..

[52] Peralta-Videa J.R., Rosa G.D.L., Gonzalez J.H., Gardea-Torresdey J.L. (2004). Effects of the growth stage on the heavy metal tolerance of alfalfa plants. Adv. Environ. Res..

[53] Li Y., Wei M., Wei W., Zhang W., Liu L. (2023). Feasibility of soil oxidation-reduction potential in judging shear behaviour of hydrocarbon-contaminated soil. J. Environ. Manag..

[54] Narayanan M., Ma Y. (2023). Mitigation of heavy metal stress in the soil through optimized interaction between plants and microbes. J. Environ. Manag..

[55] Rasool A., Imran Mir M., Zulfajri M., Hanafiah M.M., Azeem Unnisa S., Mahboob M. (2021). Plant growth promoting and antifungal asset of indigenous rhizobacteria secluded from saffron (*Crocus sativus* L.) rhizosphere. Microb. Pathog..

[56] Ali Q., Ayaz M., Yu C., Wang Y., Gu Q., Wu H., Gao X. (2022). Cadmium tolerant microbial strains possess different mechanisms for cadmium biosorption and immobilization in rice seedlings. Chemosphere.

[57] Kalaji H.M., Bąba W., Gediga K., Goltsev V., Samborska I.A., Cetner M.D., Dimitrova S., Piszcz U., Bielecki K., Karmowska K. (2018). Chlorophyll fluorescence as a tool for nutrient status identification in rapeseed plants. Photosynth. Res..

[58] Šimonová E., Henselová M., Masarovičová E., Kohanová J. (2007). Comparison of tolerance of Brassica juncea and Vigna radiata to cadmium. Biol. Plant..

[59] Rai R., Agrawal M., Agrawal S.B. (2016). Impact of Heavy Metals on Physiological Processes of Plants: With Special Reference to Photosynthetic System. Plant Responses Xenobiotics.

[60] Montes-Osuna N., Gómez-Lama Cabanás C., Valverde-Corredor A., Legarda G., Prieto P., Mercado-Blanco J. (2021). Evaluation of Indigenous Olive Biocontrol Rhizobacteria as Protectants against Drought and Salt Stress. Microorganisms.

[61] Zerrouk I.Z., Rahmoune B., Khelifi L., Mounir K., Plantarum J. (2019). Algerian Sahara PGPR confers maize root tolerance to salt and aluminum toxicity via ACC deaminase and IAA. Acta Physiol. Plant..

[62] Bhattacharjee S. (2014). Membrane lipid peroxidation and its conflict of interest: The two faces of oxidative stress. Curr. Sci..

[63] Møller I.M., Jensen P.E., Hansson A. (2007). Oxidative Modifications to Cellular Components in Plants. Annu. Rev. Plant Biol..

[64] Ranf S., Gisch N., Schäffer M., Illig T., Westphal L., Knirel Y.A., Sánchez-Carballo P.M., Zähringer U., Hückelhoven R., Lee J. (2015). A lectin S-domain receptor kinase mediates lipopolysaccharide sensing in *Arabidopsis thaliana*. Nat. Immunol..

[65] Molinaro A., Newman M.-A., Lanzetta R., Parrilli M. (2009). The Structures of Lipopolysaccharides from Plant-Associated Gram-Negative Bacteria. Eur. J. Org. Chem..

[66] Shang-Guan K., Wang M., Nang M.P.S.H., Li P., Li Y., Qi F., Zhang D., Cao M., Kim C., Weng H. (2018). Lipopolysaccharides Trigger Two Successive Bursts of Reactive Oxygen Species at Distinct Cellular Locations. Plant Physiol..

[67] Kumar V., Umrao P.D., Kaistha S.D., Bauddh K., Ma Y. (2022). Chapter12—Beneficial plant microbiome assisted chromium phytoremediation. Advances in Microbe-Assisted Phytoremediation of Polluted Sites.

[68] Badr N., Fawzy M., Al-Qahtani K.M. (2012). Phytoremediation: An Ecological Solution to Heavy-Metal-Polluted Soil and Evaluation of Plant Removal Ability. World Appl. Sci. J..

