# Supplementary material for: The Isolation of Lead-Tolerant PGPR from Red Clover Soil and Its Role in Promoting the Growth of Alfalfa
Source: Microorganisms. 2025 Jan 19;13(1):210. doi: 10.3390/microorganisms13010210 (PMC11767498; doi:10.3390/microorganisms13010210)
Supplement: Supplementary file 1 [file microorganisms-13-00210-s001.zip › microorganisms-3437579-supplementary.pdf]

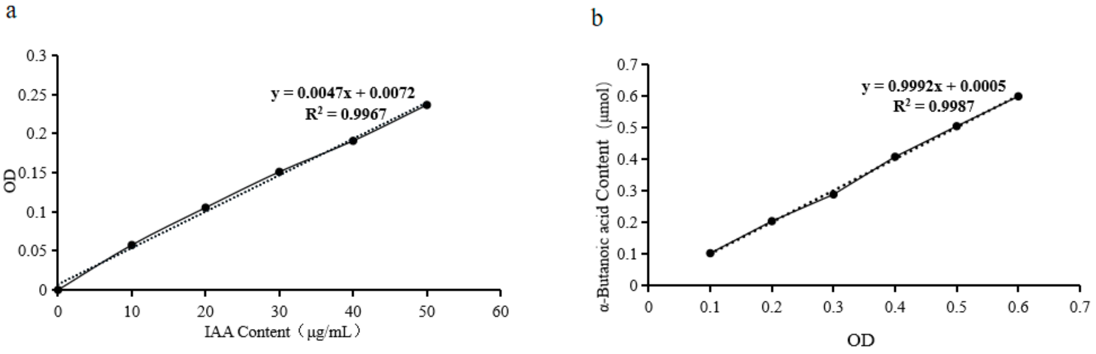

Figure S1 Standard Curve of Quantitative Rescreening of Lead-Tolerant Rhizosphere Growth-Promoting Bacteria a: IAA Standard Curve. IAA standard solutions with concentrations of 0, 10, 20, 30, 40, and 50 μg/mL were prepared and mixed with the Salkowski colorimetric solution at a volume ratio of 1:1. The OD530 value of the IAA reaction solution at each concentration was determined after the reaction was carried out at room temperature and away from light for 30 minutes (the blank control was a 1:1 mixture of distilled water and the Salkowski colorimetric solution, under the same conditions of temperature and reaction time). The IAA standard curve was made with the IAA concentration as the abscissa and the OD530 value as the ordinate. b: Standard Curve of Alpha-Butanonic Acid. A 0.1mol/L Tris-HCl (pH 8.5) buffer was employed to prepare the standard sample of alpha-butanolic acid, and the OD540 value was determined using an ultraviolet spectrophotometer to construct a standard curve of alpha-butanolic acid. The standard curve of bovine serum albumin was prepared, the total protein content in the cell extract was determined, and the ACC deaminase activity was expressed as the μmol of α-butanonic acid catalyzed by bacterial protease per milligram per hour in the reaction system, and the ACC deaminase activity of the strain was calculated.

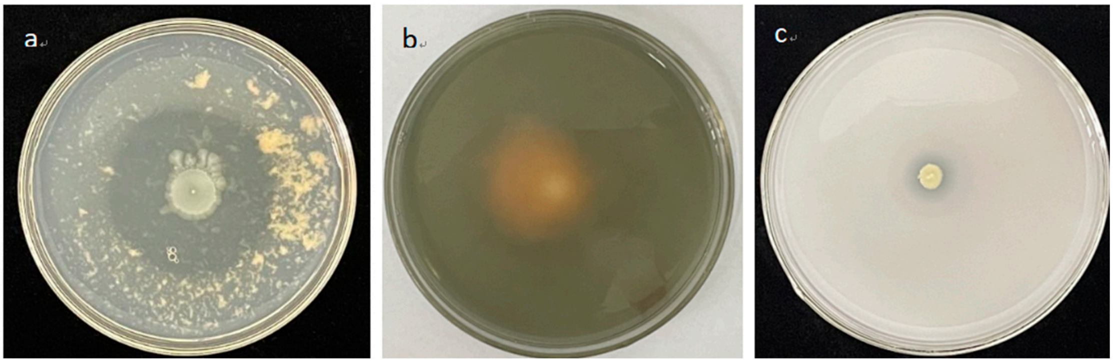

Figure S2 shows part of the positive results of the identification of growth-promoting ability.

a represents the positive result of protease, b represents the positive result of ferrisupport, and c represents the positive result of dissolved inorganic phosphorus.

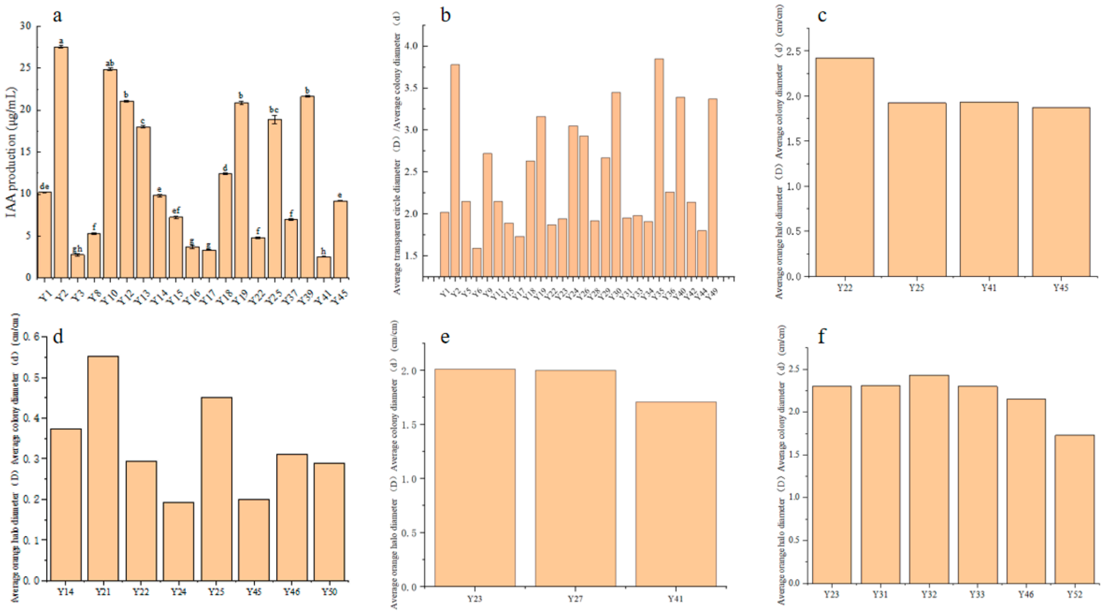

Figure S3 Quantitative rescreening of lead-resistant PGPR strains:

a: IAA production capacity of the strain; b: D/d value of the strain on protease medium; c: D/d value of the strain on inorganic phosphorus hydrolysis medium (NPA); d: ACC deaminase activity of the strain; e: D/d value of the strain on CAS detection medium; f: D/d value of the strain on Congo red medium. Different lowercase letters indicate significant differences in auxin production capacity among different strains of bacteria ($p<0.05$).

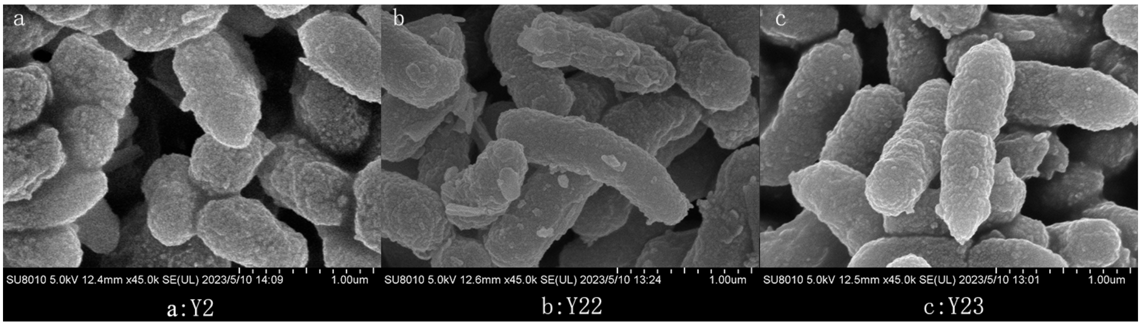

Figure S4 Scanning electron microscopy images of the three selected strains: Strain a :Y2, strain b: Y22, and strain c:Y23.

Table S1 Bacterial capacity qualitative identification table

Strain number	Auxin	Proteinase	Phosphate leaching	ACC deaminase	Siderophores	Cellulase
Y1	+	+	−	−	−	−
Y2	+	+	−	−	−	−
Y3	+	−	−	−	−	−
Y4	−	−	−	−	−	−
Y5	−	+	−	−	−	−
Y6	−	+	−	−	−	−
Y7	−	−	−	−	−	−
Y8	+	−	−	−	−	−
Y9	−	+	−	−	−	−
Y10	+	−	−	−	−	−
Y11	−	+	−	−	−	−
Y12	+	−	−	−	−	−
Y13	+	−	−	−	−	−
Y14	+	−	−	+	−	−
Y15	+	+	−	−	−	−
Y16	−	−	−	−	−	−
Y17	+	+	−	−	−	−
Y18	+	+	−	−	−	−
Y19	+	+	−	−	−	−
Y20	−	−	−	−	−	−
Y21	−	−	−	+	−	−
Y22	+	+	+	+	−	−
Y23	−	+	−	−	+	+
Y24	−	+	−	+	−	−
Y25	+	−	+	+	−	−
Y26	−	+	−	−	−	−
Y27	+	−	−	−	+	−
Y28	−	+	−	−	−	−
Y29	−	+	−	−	−	−
Y30	−	+	−	−	−	−
Y31	−	+	−	−	−	+
Y32	−	−	−	−	−	+
Y33	−	+	−	−	−	+
Y34	−	+	−	−	−	−
Y35	−	+	−	−	−	−
Y36	−	+	−	−	−	−
Y37	+	−	−	−	−	−
Y38	−	−	−	−	−	−
Y39	+	−	−	−	−	−
Y40	−	+	−	−	−	−
Y41	−	−	+	−	+	−

Y42	−	+	−	−	−	−
Y43	−	−	−	−	−	−
Y44	−	+	−	−	−	−
Y45	+	−	+	+	−	−
Y46	−	−	−	+	−	+
Y47	−	−	−	−	−	−
Y48	−	−	−	−	−	−
Y49	−	+	−	−	−	−
Y50	−	−	−	+	−	−
Y51	−	−	−	−	−	−
Y52	−	−	−	−	−	+

Table S2 Growth promotion ability table of tested strains

Strain number	Auxin	Proteinase	Phosphate leaching	ACC deaminase	Siderophores	Cellulase
Y2	+	+	−	−	−	−
Y22	+	+	+	+	−	−
Y23	−	+	−	−	+	+

Table S3 Effects of strains on radicle length of alfalfa seeds under different concentrations of lead stress

Treatment	Lead concentration（mg/L）					
	0	250	500	1000	2500	5000
CK	2.60±0.61Aa	2.60±0.26Aa	1.43±0.11Bb	0.88±0.06BCa	0.58±0.06CDc	0.26±0.06Dc
Y2	2.88±0.62Aa	2.28±0.72Aa	2.45±0.23Aa	1.10±0.17Ba	0.95±0.04Ba	0.65±0.04Ba
Y22	2.73±0.47Aa	2.48±0.28Aa	2.43±0.21Aa	0.95±0.04Ba	0.65±0.08BCbc	0.38±0.08CBc
Y23	2.83±0.23Aa	2.60±0.19Aa	1.73±0.15Bab	1.05 ±0.07Ca	0.66±0.06Dbc	0.50±0.06Db

These values are mean ± standard deviation, and lowercase letters indicate that under different lead stress concentrations, the application of bacterial strains has a significant ($p<0.05$) effect on the embryonic root length of alfalfa seeds.

Table S4 Effects of strains on alfalfa seed germ growth under different concentrations of lead stress

Treatment	Lead concentration（mg/L）					
	0	250	500	1000	2500	5000
CK	2.38±0.19Aa	2.38±0.18Aa	2.08±0.35Aa	1.98±0.08Aa	0.60±0.08Bc	0.58±0.08Bc
Y2	2.2±0.25ABCa	2.33±0.29ABa	2.50±0.21Aa	2.35±0.11ABa	1.93±0.06BCa	1.83±0.06Ca
Y22	2.40±0.25Aa	2.68±0.11Aa	2.48±0.11Aa	2.30±0.06Aa	1.40±0.08Bb	1.00±0.08Bb
Y23	2.55±0.15Aa	2.55±0.27Aa	2.08±0.08Ba	2.38± 0.04ABa	1.28±0.08Cb	0.75±0.08Dbc

These values are mean ± standard deviation, and lowercase letters indicate that the application of the strain has a significant effect on the seed germ growth of alfalfa seeds under different lead stress concentrations ($p<0.05$).

Table S5 The gene sequences of Y2, Y22, and Y23

Strain number	GenBank number	Gene sequences
Y2	PQ788471	GGAACGTATTCACCGCGACATTCTGATTCGCGATTACTAGCGA TTCCGACTTCACGCAGTCGAGTTGCAGACTGCGATCCGGACTA CGATCGGTTTTATGGGATTGGCTCCACCTCGCGGCTTGGCAAC CCTCTGTACCGACCATTGTAGCACGTGTGTAGCCCAGGCCGTA AGGGCCATGATGACTTGACGTCATCCCCACCTTCCTCCGGTTT GTCACCGGCAGTCTCCTTAGAGTGCCCACCATTACGTGCTGGT AACTAAGGACAAGGGTTGCGCTCGTTACGGGACTTAACCCAA CATCTCACGACACGAGCTGACGACAGCCATGCAGCACCTGTC TCAATGTTCCCGAAGGCACCAATCTATCTCTAGAAAGTTCATT GGATGTCAAGGCCTGGTAAGGTTCTTCGCGTTGCTTCGAATTA AACCACATGCTCCACCGCTTGTGCGGGCCCCCGTCAATTCATT TGAGTTTTAACCTTGCGGCCGTACTCCCCAGGCGGTCAACTTA ATGCGTTAGCTGCGCCACTAAAAGCTCAAGGCTTCCAACGGC TAGTTGACATCGTTTACGGCGTGGACTACCAGGGTATCTAATC CTGTTTGCTCCCCACGCTTTCGCACCTCAGTGTCAGTATTAGTC CAGGTGGTCGCCTTCGCCACTGGTGTTCCTTCCTATATCTACG CATTTCACCGCTACACAGGAAATTCCACCACCCTCTACCATAC TCTAGTCAGTCAGTTTTGAATGCAGTTCCCAGGTTGAGCCCGG GGATTTCACATCCAACTTAACAAACCACCTACGCGCGCTTTAC GCCCAGTAATTCCGATTAACGCTTGCACCCTCTGTATTACCGC GGCTGCTGGCACAGAGTTAGCCGGTGCTTATTCTGTCGGTAAC GTCAAAACACTAACGTATTAGGTTAATGCCCTTCCTCCCAACT TAAAGTGCTTTACAATCCGAAGACCTTCTTCACACACGCGGCA TGGCTGGATCAGGCTTTCGCCCATTGTCCAATATTCCCCACTG CTGCCTCCCGTAGGAGTCTGGACCGTGTCTCAGTTCCAGTGTG ACTGATCATCCTCTCAGACCAGTTACGGATCGTCGCCTTGGTG AGCCATTACCTCACCAACTAGCTAATCCGACCTAGGCTCATCT GATAGCGCAAGGCCCGAAGGTCCCCTGCTTTCTCCCGTAGGA CGTATGCGGTATTAGCGTTCGTTTCCGAACGTTATCCCCCACT ACCAGGCAGATTCCTAGGCATTACTCACCCGTCCGCCGCTCTC AAGAGAAGCAAGCTTCTCTCTACCGCTCGACTTGCATGTGTTA GGCCTGCCGCCAGCGTTCAATCTGAGCCAGTCAAAACTC
Y22	PQ835923	GGTGCGGCGGCTACACATGCAGTCGAGCGGTAGAGAGAAGCT TGCTTCCTCCTTGAGAGCGGCGGACGGGTGAGTAATGCCTAG GAATCTGCCTGGTAGTGGGGGATAACGTTCGGAAACGGACGC TAATACCGCATACGTCCTACGGGAGAAAGCAGGGGACCTTCG GGCCTTGCGCTATCAGATGAGCCTAGGTCGGATTAGCTAGTTG GTGGGGTAATGGCTCACCAAGGCGACGATCCGTAACTGGTCT GAGAGGATGATCAGTCACACTGGAACTGAGACACGGTCCAGA CTCCTACGGGAGGCAGCAGTGGGGAATATTGGACAATGGGCG AAAGCCTGATCCAGCCATGCCGCGTGTGTGAAGAAGGTCTTC

		GGATTGTAAAGCACTTTAAGTTGGGAGGAAGGGTTGTAGATT<br>AATACTCTGCAATTTTGACGTTACCGACAGAATAAGCACCGG<br>CTAACTCTGTGCCAGCAGCCGCGGTAATACAGAGGGTGCAAG<br>CGTTAATCGGAATTACTGGGCGTAAAGCGCGCGTAGGTGGTT<br>TGTTAAGTTGGATGTGAAATCCCCGGGCTCAACCTGGGAACT<br>GCATTCAAAACTGACTGACTAGAGTATGGTAGAGGGTGGTGG<br>AATTTCCTGTGTAGCGGTGAAATGCGTAGATATAGGAAGGAA<br>CACCAGTGGCGAAGGCGACCACCTGGACTAATACTGACACTG<br>AGGTGCGAAAGCGTGGGGAGCAAACAGGATTAGATACCCTGG<br>TAGTCCACGCCGTAAACGATGTCAACTAGCCGTTGGAAGCCTT<br>GAGCTTTTAGTGGCGCAGCTAACGCATTAAGTTGACCGCCTGG<br>GGAGTACGGCCGCAAGGTTAAAACTCAAATGAATTGACGGGG<br>GCCCGCACAAGCGGTGGAGCATGTGGTTTAATTCGAAGCAAC<br>GCGAAGAACCTTACCAGGCCTTGACATCCAATGAACTTTCCA<br>GAGATGGATTGGTGCCTTCGGGAACATTGAGACAGGTGCTGC<br>ATGGCTGTCGTCAGCTCGTGTCGTGAGATGTTGGGTTAAGTCC<br>CGTAACGAGCGCAACCCTTGTCCTTAGTTACCAGCACGTAATG<br>GTGGGCACTCTAAGGAGACTGCCGGTGACAAACCGGAGGAAG<br>GTGGGGATGACGTCAAGTCATCATGGCCCTTACGGCCTGGGC<br>TACACACGTGCTACAATGGTCGGTACAGAGGGTTGCCAAGCC<br>GCGAGGTGGAGCTAATCCCATAAAACCGATCGTAGTCCGGAT<br>CGCAGTCTGCAACTCGACTGCGTGAAGTCGGAATCGCTAGTA<br>ATCGCGAATCAGAATGTCGCGGTGAATACGTTCCCGGGCCTT<br>GTACACACCGCCCGTCACACCATGGGAGTGGGTTGCACCAGA<br>AGTAGCTAGTCTAACCTTCGGGAGGACGGTACCACGATGATA<br>CG
Y23	PQ788473	TCCAGCTTCATGTAGGCGAGTTGCAGCCTACAATCCGAACTGA<br>GAACGGTTTTATGAGATTAGCTCCACCTCGCGGTCTTGCAGCT<br>CTTTGTACCGTCCATTGTAGCACGTGTGTAGCCCAGGTCATAA<br>GGGGCATGATGATTTGACGTCATCCCCACCTTCCTCCGGTTTG<br>TCACCGGCAGTCACCTTAGAGTGCCCAACTTAATGATGGCAA<br>CTAAGATCAAGGGTTGCGCTCGTTGCGGGACTTAACCCAACA<br>TCTCACGACACGAGCTGACGACAACCATGCACCACCTGTCAC<br>TCTGCTCCCGAAGGAGAAGCCCTATCTCTAGGGTTTTCAGAGG<br>ATGTCAAGACCTGGTAAGGTTCTTCGCGTTGCTTCGAATTAAA<br>CCACATGCTCCACCGCTTGTGCGGGCCCCCGTCAATTCCTTTG<br>AGTTTCAGCCTTGCGGCCGTACTCCCCAGGCGGAGTGCTTAAT<br>GCGTTAACTTCAGCACTAAAGGGCGGAAACCCTCTAACACTT<br>AGCACTCATCGTTTACGGCGTGGACTACCAGGGTATCTAATCC<br>TGTTTGCTCCCCACGCTTTCGCGCCTCAGTGTCAGTTACAGAC<br>CAGAAAGTCGCCTTCGCCACTGGTGTTCCTCCATATCTCTACG<br>CATTTCACCGCTACACATGGAATTCCACTTTCCTCTTCTGCACT<br>CAAGTCTCCCAGTTTCCAATGACCCTCCACGGGTTGAGCCGTG<br>GGCTTTCACATCAGACTTAAGAAACCACCTGCGCGCGCTTTAC

GCCCAATAATTCCGGATAACGCTTGCCACCTACGTATTACCGC
GGCTGCTGGCACGTAGTTAGCCGTGGCTTTCTGGTTAGGTACC
GTCAAGGTGCCAGCTTATTCAACTAGCACTTGTTCTTCCCTAA
CAACAGAGTTTTACGACCCGAAAGCCTTCATCACTCACGCGG
CGTTGCTCCGTCAGACTTTCGTCCATTGCGGAAGATTCCCTAC
TGCTGCCTCCCGTAGGAGTCTGGGCCGTGTCTCAGTCCCAGTG
TGGCCGATCACCCTCTCAGGTCGGCTACGCATCGTTGCCTTGG
TGAGCCGTTACCTCACCAACTAGCTAATGCGACGCGGGTCCAT
CCATAAGTGACAGCCGAAGCCGCCTTTCAATTTCGAACCATGC
GGTTCAAAATGTTATCCGGTATTAGCCCCGGTTTCCCGGAGTT
ATCCCAGTCTTATGGGCAGGTTACCCACGTGTTACTCACCCGT
CCGCCGCTAACTTCATAAGAGCAAGCTCTTAATCCATTCGCTC
GACTTGCATGTATTAGGCACGCCGCCAGCGTTCATCCTGAGCC
AGATTCAAAACTCTA
